# *Salmonella* Modulates B Cell Biology to Evade CD8^+^ T Cell-Mediated Immune Responses

**DOI:** 10.3389/fimmu.2014.00586

**Published:** 2014-11-21

**Authors:** Marcela Lopez-Medina, Araceli Perez-Lopez, Celia Alpuche-Aranda, Vianney Ortiz-Navarrete

**Affiliations:** ^1^Departamento de Biomedicina Molecular, Centro de Investigación y Estudios Avanzados del IPN, México City, DF, Mexico; ^2^Department of Microbiology and Molecular Genetics, Irvine School of Medicine, University of California, Irvine, CA, USA; ^3^Instituto Nacional de Salud Pública, Secretaría de Salud y Asistencia, Cuernavaca, Morelos CP, Mexico

**Keywords:** B cells, *Salmonella*, CD8 T cells, cross-presentation, PD-L1

## Abstract

Although B cells and antibodies are the central effectors of humoral immunity, B cells can also produce and secrete cytokines and present antigen to helper T cells. The uptake of antigen is mainly mediated by endocytosis; thus, antigens are often presented by MHC-II molecules. However, it is unclear if B cells can present these same antigens via MHC-I molecules. Recently, *Salmonella* bacteria were found to infect B cells, allowing possible antigen cross-processing that could generate bacterial peptides for antigen presentation via MHC-I molecules. Here, we will discuss available knowledge regarding *Salmonella* antigen presentation by infected B cell MHC-I molecules and subsequent inhibitory effects on CD8^+^ T cells for bacterial evasion of cell-mediated immunity.

## Introduction

*Salmonella typhi* is the causative agent of typhoid fever in human beings, while infection with *Salmonella enterica* serovar Typhimurium (*Salmonella typhimurium*) produces a systemic illness in mice similar to that in human beings (1). In susceptible mice, the bacteria reside inside *Salmonella*-containing vacuoles (SCVs) of neutrophils, macrophages, and dendritic cells, in which they replicate, resist killing, and induce systemic disease (2–5). Uptake of *Salmonella* is mediated by the coordinated action of several virulence proteins translocated through the type III secretion system (T3SS), encoded by genes of *Salmonella* pathogenicity islands (SPIs) (6). While SPI-1 genes encode T3SS translocated proteins essential during bacterial invasion, T3SS SPI-2 genes are expressed once the bacteria are within the phagosome (7).

The bacteria exploit several types of immune cells for long-term survival (8–10). To survive within these cells and promote colonization, the bacteria release several virulence proteins that alter host cell functions, such as cytoskeletal architecture, membrane trafficking, signal transduction, cell death, cell trafficking, and cytokine gene expression (5, 6). This review focuses on the role of B cells during *Salmonella* infection, specifically as a niche from which the bacteria can evade immune responses and survive long-term within the host.

## General Antigen Processing and Presentation

Antigen location influences its proteolytic processing pathway and its access to different classes of MHC molecules. Subsequent presentation of these antigens by MHC-I or MHC-II molecules is necessary to induce a T cell immune response. Extracellular antigens are captured by antigen-presenting cells (APCs) through phagocytosis, macropinocytosis, or endocytosis. Newly formed phagosomes containing antigen undergo progressive trafficking characterized by acquiring or losing endosomal markers to generate a mature phagosome. Finally, their fusion with lysosomes allows complete degradation of their cargo due mainly to serine proteases (cathepsins) (11). Assembly of peptide/MHC-II complexes takes place in a multilamellar endosomic compartment that contains newly synthesized MHC-II molecules bound with invariant chain-peptide (CLIP) and machinery necessary for efficient peptide loading. The acidic environment facilitates the exchange of CLIP for antigenic peptide, catalyzed by H-2M in mice or HLA-DM in human beings. Recycled MHC-II molecules from the cell surface can also be used to form peptide-MHC-II complexes. Then, the peptide-MHC-II complexes newly formed are transported to the plasma membrane. Finally, effective MHC-II presentation requires clustered peptide/MHC-II complexes at the APC surface that can subsequently interact with the T cell receptor (TCR) and CD4 co-receptor (11, 12).

Alternately, intracellular antigens in the majority of cells are processed within the cytosol by proteosomal degradation. The peptide fragments are then translocated to the endoplasmic reticulum (ER) lumen by the transporter associated with presentation. Nascent MHC-I molecules and β2-microglobulin associate with the ER proteins tapasin, calreticulin, and Erap57, which allows glycosylation of MHC-I and optimal folding necessary after peptide binding. Then newly peptide/MHC-I complexes are transported to the cell surface (12, 13). Stable heterotrimeric complexes are necessary to engage the TCR and CD8 co-receptor. However, extracellular antigens localized in vesicular compartments of APCs can also be efficiently presented by MHC-I molecules (14), a process known as cross-presentation or cross-priming. At least four routes for cross-priming have been described (15): (1) the cytosolic route requires peptide translocation from the phagosomes to the cytosol for their proteosomal processing and subsequent ER translocation (16); (2) the vacuolar route involves peptides generated within the phagosome be loaded in intravacuolar-recycled MHC-I molecules (17); (3) the antigen is cross-processed through a phagosome-cytosol-phagosome alternating pathway (18); and (4) peptides are processed in a previously non-characterized endocytic compartment, secreted into the cytosol, and loaded onto empty MHC-I molecules on the surfaces of macrophages and bystander cells (19, 20).

## *Salmonella* Interferes with Antigen-Processing Mechanisms

*Salmonella* evade acquired immune responses to establish a chronic infection (21, 22). T cell responses can be inhibited by impaired APC antigen processing and presentation caused by bacterial proteins encoded by SPI-2 genes. As mentioned previously, *Salmonella* interferes with normal cell trafficking; for example, *Salmonella* protein SpiC inhibits maturation of *Salmonella*-containing phagosomes into phagolysosomes in macrophages and dendritic cells (2, 23–25). In addition, the phosphoinositide phosphatase SopB modulates vesicular trafficking (26). This virulence protein manipulates membrane surface charges of nascent SCVs by reducing levels of the negatively charged lipids phosphatidylinositol-4-5-biphosphate and phosphatidylserine, thus resulting in SCV maturation (27). Inhibition of phagosome acidification has been observed in macrophage cell lines (e.g., IC21) and may impede the proteolitic activity of cathepsins residing in late-endosomal compartments; this mechanism could also modify peptide processing prior to presentation (28). The integrity of the SCV, attributed to SifA, is also crucial for its resistance to oxidative killing mediated by the phagocyte oxidase phox (2). *Salmonella* mutants defective in SPI-1 and SPI-2 genes show reduced proliferation within macrophages, indicating these gene products could limit the source of peptides for antigen presentation, resulting in delayed T cell responses (29, 30). In support of this finding, Helaine et al. recently used fluorescent dilution to study intracellular replication of bacteria to determine the vacuolar environment induces phenotypic heterogeneity, thereby explaining the presence of non-replicating, yet persistent, *Salmonella* that could provide a reservoir for relapsing infection (31). Additionally, studies in human beings reveal the bacteria can control surface MHC-II expression through ubiquitination (32). Thus, *Salmonella* can impair antigen processing and presentation steps at multiple levels to prevent activation of T cell responses.

## *Salmonella* Evade T Cell Responses

An immunosuppressive effect on T cells, both dependent and independent of bacteria, has been observed during *Salmonella* infection. Basel Al-Ramaldi first noted this effect in macrophages infected with an attenuated strain of *Salmonella* cultured with splenocytes in transwell plates. Soluble factors mediated T cell suppression, but the exact nature of the factor(s) was not determined at that time (33). Later, T cell proliferation assays were performed in the presence or absence of the inducible nitric oxide synthase (iNOS) inhibitor l-NMMA, which showed the suppression is also mediated by dendritic cells and is dependent on NOS induction (34). When nitric oxide was blocked with aminoguanidine, the inhibition of T cell suppression, macrophage activity, and polymorphonuclear leukocyte influx was observed (35). Thus, nitric oxide may play multiple biological roles during *Salmonella* infection. Other studies employing the human-restricted strain *S. typhi* showed the polysaccharide Vi, released from *Salmonella*, leads to an impairment of IL-2 production in T cells by interacting with the membrane prohibitin complex (36). Other transwell assays with CD8^+^ T cells and dendritic cells infected with *Salmonella* deficient in the proteins SPI-1, SPI-2, phoP, and sti or carrying virulence plasmids demonstrated priming can be inhibited by direct contact with the bacteria (37). Moreover, exposure to LPS during priming in *Salmonella-*infected mice suppressed IL-2 and TNF-α production of flagellin-specific CD4^+^ T cells, resulting in exacerbation of murine typhoid (38).

Other mechanisms for T cell evasion *Salmonella* infection have been described. Experiments involving adoptive transfer of CD4^+^ T cells from TCR-transgenic mice into *Salmonella*-infected mice showed the bacteria induce a progressive culling of newly activated, high-avidity, antigen-specific CD4^+^ T cells that express higher levels of programed death-ligand 1 (PD-L1) in an SPI-2 dependent manner (39, 40). This mechanism reshapes the repertoire of antigen-specific T cells after *Salmonella* infection. Furthermore, several groups have found the bacteria are able to reach the thymus (41, 42). We have observed that infections of the thymus cause Vβ chain rearrangements of TCRs in single-positive CD8^+^ T cells, possibly leading to a biased selection of certain types of clonal cell populations (unpublished data). *Salmonella* can downregulate TCR expression by reducing the amount of both surface and intracellular TCR-β chain in T cells co-cultured with *S. typhimurium* (43). However, it is unknown if the bacteria could trigger or produce crosstalk between signaling pathways that would lead to this phenotype.

Because regulatory T (T_reg_) cells mediate immune suppression, these cells can play both detrimental and protective roles in host defense against infection. Johanns et al. has shown that suppressive capacity of Treg coincide with a delay of elicing protective response during early *Salmonella* infection, contrary during late infection Treg suppressive potency diminish (44). Moreover, peritoneal NK1.1 αβ T cells reduced IL-12 production in macrophages by secretion of IL-4 upon TCR activation, during the early phase of *Salmonella* infection (45). Thus, *Salmonella* employ several strategies to overcome acquired immunity in order to persist and produce a chronic infection in the host.

## B Cells as APCs in T Cell Priming

The introduction of fluorescence-activated cell sorting (FACS) revolutionized the study of B cells, allowing the classification of B cells from lymph nodes, the spleen, and more recently, from the liver (46) into phenotypically and functionally distinct populations, denoted B1 and B2. The B2 lymphocytes are further subdivided into marginal zone B (MZ-B) and follicular B (FO-B) cells, while B1 lymphocytes are grouped as B1a or B1b cells. All subsets differ in their development, location, function, and most importantly, their ability to present antigens to T cells. For B cells to become competent APCs, they first must receive signals either from the B cell receptor (BCR) or Toll-like receptors (TLRs) for activation. This feature allows enhanced B cells uptake of both soluble and particulate (phagocytosed) antigens, followed by the expression of co-stimulatory molecules and the subsequent processing and presentation of antigens with MHC-I or MHC-II molecules (47).

Marginal zone B cells are strategically located in the bloodstream for easy activation and to intercept and react to blood-borne antigens (48, 49). Antigens captured by MZ-B cells are delivered to follicular dendritic cells through shuttling dependent on the CXCR5-S1P_1_-S1P_3_ axis (50). However, MZ-B cells can also initiate a rapid first line of defense, demonstrated by Olivier et al., in which they express higher basal levels of co-stimulatory molecules CD80 and CD86, which are rapidly upregulated within 6–24 h after LPS exposure or BCR signaling. In fact, LPS-stimulated MZ-B cells induced a vigorous proliferation of alloreactive T cells *in vitro*, in contrast with LPS-stimulated FO-B cells, which then developed into mature plasma cells (51). In another set of experiments, Attanavanich et al. demonstrated that *in vivo* hen egg lysozyme (HEL)-specific MZ-B cells are more potent activators of naïve TCR-transgenic CD4^+^ T cells than HEL-specific FO-B cells. The MZ-B cells likely have better access to the antigen and can rapidly migrate toward the T cell area, followed by plasma cell differentiation (52). Together, these experiments highlight the role of MZ-B cells to provide a bridge between innate and adaptive immune responses.

Similarly, B1 cells express higher basal levels of CD80 and CD86, suggesting their potential role in rapidly initiating a T cell response (53). The capability of peritoneal cavity B1 cells to phagocytose, process, and present particulate antigens, such as OVA bound to latex beads (1 μM) (54). Interestingly, MZ-B cells, in conjunction with B1 cells from either the spleen or peritoneal cavity, participate in the response against blood-borne antigens (55). In addition to MZ-B and B1 cells, parabiosis studies suggest that mature B cells located in the perisinusoidal niche of bone marrow, which have access to the circulatory system and can freely enter and exit the bone marrow, are also specialized for T cell-independent responses to blood-borne antigens (56). Previous paradigms describing B cell antigen presentation have changed, further supported by recent findings involving phagocytic IgM^+^ cells from teleost fish and amphibians that indicate an evolutionary relationship between B cells and macrophages (57). This theory suggests B cells may have evolved from ancient phagocytic cells to macrophage-like cells to B cells that maintained their ability to phagocytose. Therefore, when B cells are activated, they become potent APCs when they encounter specific antigens, leading to cognate T-B cell interactions, T cell activation, and germinal center (GC) reactions. The amount of antigen captured and presented by GC B cells to follicular helper T (T_fh_) cells is proportional to cell division and hypermutation rates because GC B cells with the highest affinity for antigens are selectively expanded and diversified (58).

In addition to priming T cells, APCs can also provide signals that instruct T cells to enter into effector/memory differentiation programs. Soo Choi et al. found that T_fh_ differentiation is mediated by two key players; during priming, dendritic cells induce Bcl6 expression in T_fh_ cells, while the stable commitment to this differentiation program requires interaction with FO-B cells (59). This mechanism was explored by experiments in which antigen-specific T cells from MD4/μMT B cell-deficient mice showed reduced levels of Bcl6 expression at day 7 post-immunization against lymphocytic choriomeningitis virus (LCMV). Experiments using B cell/dendritic cell MHC-II-deficient mice reinforced the role of MHC-II in antigen presentation by FO-B cells in cooperation with T_fh_ differentiation (60). This model suggested B cells participate in the initiation, maintenance, and full polarization of T_fh_ differentiation (61). Regarding T_h_1 differentiation, Barr et al. have shown that an antigen-specific IgG2c primary response is absolutely dependent on MyD88 signaling to B cells in mice immunized with T cell-dependent antigen or in mice infected with *Salmonella* (62). They also found that B cell-intrinsic MyD88 signaling is required for primary effector T_h_1 cell development, whereas antigen-specific BCR-mediated presentation is necessary for the development of T_h_1 memory cells against *Salmonella* (63). In addition, MZ-B cells participate in T_h_1 cell differentiation, and Attanavanich et al. found that, when cultured *in vivo*, HEL-primed MZ-B cells from MD4 mice with naïve CD4^+^ T cells produce large amounts of T_h_1-like cytokines and IFN-γ but low levels of IL-4, IL-5, and IL-10. This expression pattern suggests MZ-B cells also provide signals for T_h_1 cell development during the primary immune response (52). These findings emphasize the non-redundant role of B cells as programmers of CD4^+^ T cell differentiation.

The ability of B cells to process and present viral antigens to CD8^+^ T cells via MHC-I molecules was first explored by Ciavarra et al. with proliferation and cytotoxicity assays using [^3^H]thymidine and ^51^Cr release, respectively. These experiments highlighted the efficacy of mitogen (LPS)-activated B cells in displaying target antigens on their cell surface membranes, which are efficiently recognized in a MHC-I-dependent manner by vesicular stomatitis virus-specific cytotoxic T cells (CTLs) (64). Other experiments employing mice infected with LCMV-Clone 13, a strain that causes persistent infections, showed that neutralizing antibodies are induced unless CD8^+^ T cells were depleted. This result suggests B cells might be actively infected and capable of presenting viral peptides on MHC-I molecules; thus, they may become targets for LCMV-specific CTLs (65). Subsequent studies by the same group used ^51^Cr release assays with splenocytes from LCMV-infected BALB/c (H-2^d^) mice and as target, LCMV-infected, neutralizing antibody-secreting hybridomas. Showed that CTLs lysed the infected hybridomas, because LCMV was endocytosed through the membrane-anchored neutralizing antibody receptor and are later eliminated by virus-specific CTLs (66). These results reinforced the role of B-cell as APC. Although not absolutely required, they do play a role in T cell priming, thus positively impacting the function of CD8^+^ T cells. Multiple cytokines, such as IL-2, IL-12, IL-21, IL-27, and IL-33, which are produced by CD4^+^ T cells, APCs, and non-hematopoietic cells from the T cell zone, participate in promoting effector T cell differentiation (67, 68). Liu et al. first explored B cells’ potential “helper role” for CD8^+^ T cells by evaluating the anti-influenza cytolytic activity of CD8^+^ T cells. They demonstrated that soluble factors released by B cells could replace the CD4^+^ T cell requirement to induce cytotoxic responses to influenza virus (69). These previous studies changed our view of B cells as APCs and showed they strongly influence an effective CD8^+^ T cell response against pathogens localized in the cytoplasm, such as viruses.

Evidence that B cells possess machinery to perform alternative pathways for antigen processing for CD8^+^ T cell priming has been presented in studies related to vaccine development and bacterial infections. For example, the *Mycobacterium tuberculosis* heat shock protein 70 (HSP70) is endocytosed, subjected to vacuolar processing, and forms highly immunogenic complexes with chaperoned peptides that are presented on MHC-I molecules to elicit a CD8^+^ T cell response (70). In one experiment, CpG oligodeoxynucleotides-activated B cells could uptake OVA-associated HSP70, in a CD91-dependent manner, process the fusion protein by vacuolar mechanisms and prime OVA-specific CD8^+^ T cells. In another study involving immune-stimulating complexes (ISCOMS) that induce strong MHC-I-restricted responses, HEL-specific B cells could uptake OVA-HEL-ISCOMS and then stimulate OVA-specific CD8^+^ T cell responses. This cross-presentation required endosomal acidification, proteosomal processing, and classical MHC-I/peptide transport (71). During bacterial infections, BCR-mediated internalization of *Salmonella* led to efficient antigen delivery to MHC-II antigen-loading compartments; however, when the proteosome was inhibited with MG-132, only a partial dependence on this protease was observed (72, 73). These data indicate B cells may have machinery to employ the phagosome-cytosol antigen presentation pathway. In addition, our group has shown that *Salmonella-*infected B cells cannot use the vacuolar alternative pathway that involves antigen processing in a vacuolar compartment, which is often followed by secretion and loading of antigenic peptides to MHC-I molecules on the surface of B cells and bystander cells (28). In sum, these studies show that B cells possess machinery necessary to induce a CD8^+^ T cell response against intracellular pathogens localized in vacuolar compartments.

We have thus far reviewed evidence that portrays B cells as highly competent APCs that positively impact T cell functions; however, B cells are also negative regulators of T cell responses therefore denoted as B_reg_ cells. Their inhibitory function has been associated mainly with IL-10 because this B cell derived-cytokine can protect against autoimmunity, yet increase the host’s susceptibility to infection (74). Recently, two separate studies identified an additional soluble factor that mediates regulatory functions in B cells. One study found IL-35 can induce the conversion of typical B cells into an IL-35-producing B_reg_ cell population dependent on STAT1 and STAT3, which are induced through signaling by IL-12Rβ2 and IL-27Rα. In addition, induced B cells exerted a suppressive influence on pathogenic T_h_17 and T_h_1 cells from experimental autoimmune uveitis-induced mice (75). The second study revealed that B cells, through activation of TLR4 and CD40, secrete IL-35. This study focused on B35 cell-deficient mice and found B cell-derived IL-35 is necessary for pathogenic T_h_17 and T_h_1 cell suppression in an autoimmune encephalomyelitis model. Moreover, a lack of IL-35 production by B cells led to increased activation of macrophages and CD4^+^ T_h_1 cells and favored B cells as APCs in a *Salmonella* infection model (76).

## B Cells during *Salmonella* Infection

T cells, particularly T_h_1 cells, are crucial for *Salmonella* infection control due to their IFN-γ secretion (62, 63, 77–81), while *Salmonella-*specific antibodies are required to resist secondary infection. The role of B cells as antibody-producing cells has been demonstrated using B cell-deficient mice (Igh-6^−^/^−^ or Igμ^−^/^−^) (82, 83) that were immunized with an attenuated strain of *Salmonella* and then challenged with a virulent strain; these mice could not resist the infection (84). In addition, transfer of immune serum to immunized B cell-deficient mice (Igμ^−^/^−^) 1 day prior to challenge with virulent *Salmonella* effectively reconstituted their immunity (83). Thus, antibody-producing B cells are key players during secondary bacterial infections. Beyond this well-known role, another study suggests B cells are required for priming T cell responses during bacterial infections. Notably, Ugrinovic et al. found a reduced frequency of both IFN-γ-producing CD4^+^ and CD8^+^ T cells in immunized, gene-targeted, B cell-deficient Igh-6^−^/^−^ mice. When primary B cells infected *in vitro* were cultured with *Salmonella*-specific CD4^+^ T cells from immunized mice, they induced modest proliferation compared to *in vitro*-infected bone marrow-derived macrophages (85). More recently, Nanton et al. evaluated T cell responses against *Salmonella* using B cell-deficient JhD mice, transgenic mice with B cells that could not class switch or secrete antibodies, and mice with B cells that could not class switch but were able to secrete IgM. They observed a decrease of both IFN-γ ^+^CD8^+^ or CD4^+^ T cells, which suggested antibodies are not required for an optimal *Salmonella*-induced T_h_1 response (86). Collectively, these studies show that *in vitro Salmonella-*infected B cells can moderately prime CD4^+^ T cells and somehow participate in the activation of T cell responses. Recent findings from Barr et al. using a *Salmonella* infection model demonstrated intrinsic MyD88-derived B cell signals play a role in effector T_h_1 cell differentiation (62, 63). Our group, along with others, has shown that *in vitro-*infected B cells produce IL-6 (87), which contributes to the early multistages of T_fh_ cell differentiation. Therefore, it is not surprising that B cells can also act as T cell programmers, although it is unknown if B cell infection may impair the multistage and multifactorial T_fh_ cell differentiation program.

## B Cells Prime CD8^+^ T Cells Responses during *Salmonella* Infection

Although CD4^+^ T cells are known as key players during *Salmonella* infection, the role of CD8^+^ T cell responses is less clear. Interestingly, CD8^+^ T cells participate in the eradication of bacteria during secondary *Salmonella* infections, but their role in primary infection seems contradictory (80, 88, 89). Previous evidence demonstrated a null to modest participation of CTLs in mice deficient of β2m or depleted of CD8^+^ T cells (80, 89). More recent reports focused on MHC-Ia-deficient mice (K^b^D^b^) demonstrated the role of CD8^+^ T cells during the later stages of a primary infection (88). In addition, the involvement of non-polymorphic MHC-Ib (Qa-1, HLA-E, H2-M3) during the response against *Salmonella* has recently gained attention due to their role as presentation molecules for *Salmonella* antigens (90, 91).

**Figure 1:**
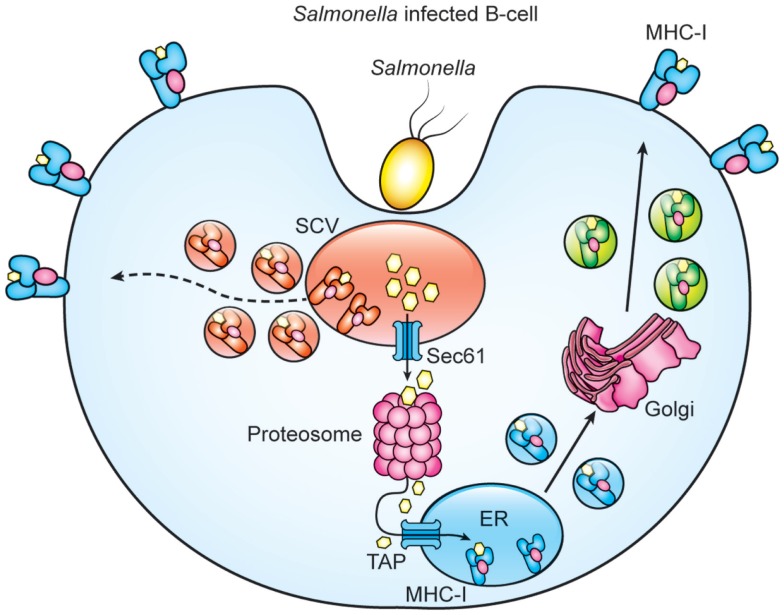
**Proteasome and paghosomal degradation are involved for cross-processing of *Salmonella* antigens by B cells**. Model of cross-priming in *Salmonella*-infected B cells. *Salmonella* infection generates antigens that are translocated to the cytosol for proteosomal processing and subsequent translocation of *Salmonella* peptides by TAPs to ER for loading MHC-I. Degradation of *Salmonella* proteins with the SCV generate peptides that load recycled MHC-I molecules and the resulting MHC-I/peptide complexes are then transported to the B cell surface.

When acting as APCs, B cells that express a *Salmonella*-specific BCR can, after bacterial internalization, reactivate human memory CD8^+^ T cells via cross-presentation, leading to a CTL response (72). However, it is unclear if primary B cells infected with *Salmonella* by the natural entry pathway could cross-process and present *Salmonella* antigens via MHC-I. By using a *Salmonella* strain (S-OVA) that expresses the OVA peptide (OVAp) fusioned with the curli protein (Crl), we evaluate whether MHC-I present *Salmonella* antigens after infection. We found that *in vitro* and *in vivo* S-OVA-infected B cells express K^b^-OVAp complexes. This presentation diminished by using inhibitors of the components of the classical (brefeldin and lactacystin) or vacuolar (leupeptin and ammonium chloride) pathways suggesting that processing of *Salmonella* antigens might involve the translocation of partial processed antigens from the SVC to the cytosol, followed by their proteosomal degradation and subsequent ER translocation (Figure 1) (unpublished data). In agreement with our results, a previous study demonstrated that, in human-specific B cells, cross-presentation of *Salmonella* antigens is partly proteosome-dependent (72). It is also likely that peptides generated within the SCV might load onto recycling MHC-I molecules (Figure 1). On the other hand, *Salmonella* infection also promotes B cell activation since expression of co-stimulatory molecules such as CD40, CD80, and CD86 within *Salmonella*-infected B cells is observed (unpublished data). In sum, these results suggest that infected B cells are capable of cross-processing and presenting *Salmonella* antigens and can express co-stimulatory molecules to become professional APCs that prime and sustain a CD8^+^ T cell response. Similarly, other studies employing antigen-specific B cells in *Salmonella* infection or diabetes type-1 models further support the capability of B cells to process and present exogenous antigens (72, 92).

## B Cells as Trojan Horses during *Salmonella* Infection

We and other groups have demonstrated that *S. typhimurium* infects and persists long-term in splenic and lymph node macrophages, splenic dendritic cells, splenic B cells, bone marrow B cell precursors, and plasma cells. Studies in human beings have shown that *S. typhi* can be isolated from bone marrow cultures, regardless of disease stage or type of pharmacological treatment (93). Bone marrow B cells present a safe niche for *Salmonella* because they cannot enter peripheral circulation until they fully mature. In human beings and mice, most *Salmonella* infections occur in the ileum, spleen, and liver (94). More recently, gallstone biofilms and the gallbladder epithelium were demonstrated niches for chronic *Salmonella* infections; however, only 3–5% of *S. typhi-*infected individuals develop a chronic infection in these sites (21, 95).

Macrophages often serve as host cells for *Salmonella* during acute and chronic infections, but the fate of these cells, as well as other infected cells, is unclear. In some cases, *Salmonella* induce host cell death, releasing the bacteria and disseminating the infection. Our group has shown that *Salmonella* inhibit pyroptosis in B cells because they abrogate IL-1β production by impairing NLRC4 transcription; thus, B cell death is not induced (87, 96). This mechanism could allow *Salmonella* survival within these cells during an innate immune response. Using the B cell line A20, we discovered the vacuolar compartment in which *Salmonella* reside is different from that in macrophages (28). Interestingly, fluorescent dilution analysis revealed that the SCV environment and nutritional deprivation of infected macrophages activate *Salmonella* virulence genes, leading to the presence of non-replicating, persistent bacteria (31). In B cells, primary infection is followed by the production of reactive oxygen species, iNOS, and pro-inflammatory cytokines IL-1β, TNF-α, and IL-6, which often control the bacteria (87). *Salmonella* replication rates within infected B cells are likely low, as we and other groups have found very few bacteria in these cells. For example, Souwer et al. used *in vitro* infection assays to determine that only 4% of human B cells phagocytose the bacteria via their BCR (73). Similarly, we have observed approximately 0.1–1% of mouse splenic primary B cells, bone marrow B cell precursors, and plasma cells get infected with *Salmonella*. However, we also discovered that after 2 months post-infection, *Salmonella* can still be isolated from infected bone marrow B cell precursors and infected plasma cells (97). Our experiments involving susceptible BALB/c mice infected with a single dose of 50 virulent *Salmonella* bacteria showed that after 1 month post-infection, bacterial CFUs could be isolated from infected splenic B1a and B1b, MZ-B, and FO-B cells (unpublished data). Thus, *Salmonella* likely exploit B cell populations to persist long-term in the host. Interestingly, if *Salmonella* infect and persist within all splenic B2 cells, infected FO-B cells, which possess migratory properties, could as act as carriers for further dissemination of *Salmonella*. Moreover, plasma cells migrate to the bone marrow and eventually undergo apoptosis (98), but these cells could also be involved in spreading the bacteria. However, it is unknown if infected splenic B cells could also differentiate into antibody-secreting cells.

The microbes’ level of persistence depends on a balance between the immune response of the host and the bacteria’s ability to survive within the cell. Certain viral pathogens (LCMV, HIV, HCV, HBV) (99–102), parasites (*Trypanosoma cruzi*, *Schistosoma mansoni*, *Tenia crassiceps*) (103–105), and some bacteria (*M. tuberculosis*, *Helicobacter pylori*, *Chlamydia trachomatis*) (106–108) can render T cell responses ineffective by benefiting from inhibitory stimuli such as PD-1:PD-L (PD-L1 and PD-L2) interactions. Some of the experiments described above showed B cells can process and present *Salmonella* antigens *in vitro* and *in vivo*. In addition, we have found that B cells remain infected long-term, suggesting they may avoid elimination by CTLs. In this context, PD-L1 and PD-L2 expression in B cells infected *in vitro* and *in vivo* with *Salmonella* was observed (unpublished data). These results suggest *Salmonella* infection provides signals that trigger the transcription of PD-L1 and PD-L2 genes. Furthermore, infected B cells likely produce both positive and inhibitory signals. These inhibitory signals may be more dominant during infection because they allow the bacteria to avoid effector CD8^+^ T cell responses. Therefore, expression of PD-L1 and PD-L2 by infected B cells could be one possible mechanism employed by the bacteria to survive within these cells and evade cell-mediated immunity. However, no current evidence indicates the PD-1:PD1-Ls axis directly terminates or attenuates CD8^+^ T cell responses during chronic *Salmonella* infection. Our current studies simply show that *Salmonella*-infected B cells express PD1-Ls during acute and chronic infections. We have also found that PD-1 is expressed on antigen-specific CD8^+^ T cells (unpublished data), so the participation of this axis during infection could explain why previous studies reported no significant CD8^+^ T cell involvement during *Salmonella* infections. Furthermore, our group has also found B1 cells can produce IL-10 when infected *in vitro* with virulent *Salmonella* (unpublished data), which, along with IL-35 production, can inhibit both innate and acquired immune responses against *Salmonella* (76, 109).

## Concluding Remarks

Many studies have sought to elucidate how *Salmonella* achieves a balance between avoiding immune responses and surviving long-term in its host. Previous research indicates *Salmonella* exploits several types of immune cells to persist during chronic infections. Here, we presented evidence that B cells are an amenable bacterial reservoir, promoting their persistence, dissemination, and evasion of CD8^+^ T cell-mediated responses. Identifying the mechanisms employed by *Salmonella*-infected B cells to avoid cell-mediated immunity is clinically significant for understanding the chronic, asymptomatic carrier stage of *Salmonella* infection that occurs in human beings following typhoid fever.

## Conflict of Interest Statement

The authors declare that the research was conducted in the absence of any commercial or financial relationships that could be construed as a potential conflict of interest.
